# Conventional agriculture and not drought alters relationships between soil biota and functions

**DOI:** 10.1038/s41598-021-03276-x

**Published:** 2021-12-14

**Authors:** Klaus Birkhofer, Andreas Fliessbach, María Pilar Gavín-Centol, Katarina Hedlund, María Ingimarsdóttir, Helene Bracht Jørgensen, Katja Kozjek, Svenja Meyer, Marta Montserrat, Sara Sánchez Moreno, Jordi Moya Laraño, Stefan Scheu, Diego Serrano-Carnero, Jaak Truu, Dominika Kundel

**Affiliations:** 1 Department of Ecology, Brandenburg University of Technology Cottbus-Senftenberg, Konrad-Wachsmann-Allee 6, 03046 Cottbus, Germany; 2 Department of Soil Sciences, Research Institute of Organic Agriculture (FiBL), 5070 Frick, Switzerland; 3 Estación Experimental de Zonas Áridas, EEZA-CSIC, 04120 Almería, Spain; 4 Department of Biology, Lund University, 22362 Lund, Sweden; 5 J.F. Blumenbach Institute of Zoology and Anthropology, Animal Ecology, University of Göttingen, 37073 Göttingen, Germany; 6 Instituto de Hortofruticultura Subtropical y Mediterránea “La Mayora” - Universidad de Málaga- Consejo Superior de Investigaciones Científicas, Málaga, Spain; 7 Department of Environment and Agronomy, National Center Institute for Agricultural and Food Research and Technology, Madrid, Spain; 8 Centre of Biodiversity and Sustainable Land Use, University of Göttingen, 37077 Göttingen, Germany; 9 Institute of Molecular and Cell Biology, University of Tartu, 51010 Tartu, Estonia; 10 Max Planck Institute of Animal Behavior, 78315 Radolfzell, Germany; 11 Department of Biology, University of Konstanz, 78464 Konstanz, Germany

**Keywords:** Agroecology, Biodiversity, Climate-change ecology, Ecosystem services

## Abstract

Soil biodiversity constitutes the biological pillars of ecosystem services provided by soils worldwide. Soil life is threatened by intense agricultural management and shifts in climatic conditions as two important global change drivers which are not often jointly studied under field conditions. We addressed the effects of experimental short-term drought over the wheat growing season on soil organisms and ecosystem functions under organic and conventional farming in a Swiss long term trial. Our results suggest that activity and community metrics are suitable indicators for drought stress while microbial communities primarily responded to agricultural practices. Importantly, we found a significant loss of multiple pairwise positive and negative relationships between soil biota and process-related variables in response to conventional farming, but not in response to experimental drought. These results suggest a considerable weakening of the contribution of soil biota to ecosystem functions under long-term conventional agriculture. Independent of the farming system, experimental and seasonal (ambient) drought conditions directly affected soil biota and activity. A higher soil water content during early and intermediate stages of the growing season and a high number of significant relationships between soil biota to ecosystem functions suggest that organic farming provides a buffer against drought effects.

## Introduction

Agricultural soils are fundamentally important habitats for terrestrial biodiversity and provide some of the most crucial ecosystem services to humanity^1^. Effects of anthropogenic global change on soil ecosystems and their biodiversity therefore receive considerable attention in the International Decade of Soils (2015–2024; International Union of Soil Science). Major anthropogenic activities, such as agricultural intensification or climate change, contribute to a loss of soil biota and related ecosystem functions and services worldwide^2–4^. Soils with high biodiversity hold the potential to provide multiple ecosystem functions simultaneously and a loss of soil biodiversity may threaten soil multifucntionality^5,6^. A high biodiversity in agricultural soils may for example contribute to the joint provision of food quantity and nutritional quality^7^.

Land-use change is partly driven by worldwide agricultural intensification aiming to satisfy the demands of a growing human population, but causing a severe degradation of arable soils^8^. Key components of agricultural intensification are an increasing use of synthetic fertilizers and chemical pesticides with well-known negative consequences for soil biota^2^. Organic agriculture is not based on the application of synthetic fertilizers and chemical pesticides. This farming system is therefore proposed as an alternative strategy to conventional agriculture with reduced environmental impact^9^, benefits for biodiversity^10^ and higher levels of ecosystem service provision^11^. Bacterial and fungal diversity, for example, respond positively to the application of organic fertilizers, while long-term application of synthetic fertilizers reduces the diversity of certain soil biota groups^12^.

Effects of land-use change on biodiversity and ecosystem functions may be aggravated by climate change^13^. Climate models predict reduced terrestrial water storage as a consequence of more severe droughts^14^ and an increasing future need for agricultural irrigation in Europe^15^. The predicted drought events will have severe effects on soil biota and associated ecosystem functions^2^ with a recent joint IPCC and IPBES workshop report emphasizing the potential negative consequences for human well-being^16^. Experimental drought, for example, negatively affects the abundance and diversity of soil biota in forests, but not necessarily in grass^17^ and heathlands^18^. These results suggest a certain level of resistance to drought in some ecosystems like grasslands^19^. However, negative effects of reduced precipitation are persistent for several groups of soil biota (e.g. microbes^20^) and soil functions^21,22^ independent of the ecosystem. The strength of drought effects partly depends on the drought intensity and duration^23^. In general, however, extended drought periods through the crop growing season are expected to severely reduce cereal yields in Europe^24^ and pose a risk to future food security^25^.

Effects of land-use intensification and climate change on soil biota and associated ecosystem functions may depend on each other^26^. It is, for example unknown, if certain farming systems provide a buffer against negative consequences of drought conditions on soil organisms and functions in agricultural soils. A high soil organic carbon (SOC) content of agricultural fields can improve the water-holding capacity, aggregate structure and water infiltration in agricultural soils^27,28^. These properties may subsequently enhance the ability of soils to store water for crop production during periods of limited precipitation^29^. Organic agriculture has been shown to result in soils with higher SOC content in (i) our study plots (conventional: 1.27% vs. organic: 1.60%^30^), (ii) the DOK agricultural long-term trial in general^31^ and (iii) agricultural top soils worldwide^32^ compared to conventional agriculture. These links between agricultural management and drought exemplify the importance to consider agricultural practices when assessing the impact of summer drought on soil biota and functions in agroecosystems^30^.

Land-use intensification and climate change may also alter the relationships between soil biota and ecosystem functions, potentially affecting synergies and disrupting relationships between biodiversity and ecosystem functions^33^. Trade-offs or synergies between biodiversity and ecosystems functions are common in the aboveground compartment of agroecosystems^34^ and are affected by local management decisions^35,36^. Agricultural management, such as fertilization and pest management practices, alter the activity, diversity, abundance and composition of belowground communities^37,38^. The expected effects on service-providing organisms in agricultural soils then hold the potential to change levels of multiple ecosystem functions and services in soils^39,40^. There is evidence for synergies between soil biodiversity and multiple ecosystem functions under certain agricultural practices (e.g. agricultural diversification^41^), but context-dependent trade-offs (e.g. negative relationships between soil biodiversity and functions) have also been reported^42,43^. Parallel to trade-offs and synergies caused by agricultural management decisions, climate change also holds the potential to alter relationships between biota and multiple ecosystem functions in soil^44^. The utilization of synergies between soil biodiversity and ecosystem functions and services in agricultural soils is key to sustainable agricultural management in the future^43^. Optimizing the benefits derived from soil processes by promoting synergies between soil biodiversity and ecosystem functions will improve food security at reduced environmental impact^42^.

We lack a general understanding of joint effects of land-use intensification and climate change on soil biota and ecosystem functions and their complex relationships^32,45^. To address this knowledge gap, we studied the individual and interactive effects of experimental short-term drought at three sampling dates during the growing season in replicated conventionally and organically managed winter wheat plots. We first analysed how two major aspects of global change, i.e.: (1) land-use intensification (conventional vs. organic agriculture that differ in fertilisation and pest management) and (2) experimental (65% precipitation reduction with rainout shelters vs controls) and ambient (reduced precipitation over the growing season) drought conditions, affect the diversity and abundance of soil biota and indicators of related ecosystem functions (for details Table 1). We then assessed how the relationships between these variables, in terms of trade-offs and synergies, were altered by both global change aspects. We hypothesize that (1) intensive conventional agriculture and drought conditions (experimental and ambient) both directly reduce the abundance and diversity of soil biota and cause lower levels of soil ecosystem functions with lowest values in the joint treatment and that (2) organic agriculture acts as a buffer against negative effects of drought conditions compared to conventional agriculture. Finally, we hypothesize that (3) relationships between soil biota and ecosystem functions are weaker or disrupted in more intensively managed conventional systems and under experimental drought conditions.

**Table 1:** Abundance- (#1–13), diversity- (Shannon index with exponential log base, #14–18) and process-related (#19–26) dependent variables in this study with unit, range, mean ± standard deviation (SD) and method.

#	Variable	Unit	Range	Mean ± SD	Method
1	Arbuscular mycorrhizal fungi (AMF) biomass	nmol/g soil	3.0–30.0	8.6 ± 6.0	Lipid extractions from soil
2	Bacterial biomass	nmol/g soil	23.0–48.8	36.9 ± 7.6	Lipid extractions from soil
3	Fungal biomass	nmol/g soil	0.7–2.2	1.2 ± 0.3	Lipid extractions from soil
4	Microbial nitrogen (N)	µgN_mic_/g dry soil	22.4–91.8	61.5 ± 20.8	Chloroform fumigation extraction
5	Microbial carbon (C)	µgC_mic_/g dry soil	158–539	386.1 ± 110.7	Chloroform fumigation extraction
6	Nematoda abundance	individuals/100 g dry soil	267.9–5604.3	1191.3 ± 821.8	Baermann funnel method
7	Collembola abundance	individuals per sample	0–70,656	9692.4 ± 16,037.0	Heat gradient extraction
8	Oribatida abundance	individuals per sample	308–20,636	2338.8 ± 2666.0	Heat gradient extraction
9	Chilopoda abundance	individuals per sample	0–252	55.2 ± 54.4	Heat gradient extraction
10	Diplopoda abundance	individuals per sample	0–1176	85.6 ± 191.1	Heat gradient extraction
11	Araneae activity density	individuals per sample	1–29	11.3 ± 5.8	Pitfall traps
12	Staphylinidae activity density	individuals per sample	0–37	6.2 ± 7.3	Pitfall traps
13	Arable weed cover	% cover	0–90	15.6 ± 23.4	Visual estimate
14	Bacterial diversity	Shannon index on OTU level	6.7–6.9	6.8 ± 0.1	16S rRNA sequencing
15	Nematoda diversity	Shannon index on genus level	1.3–2.4	1.9 ± 0.2	Baermann funnel method
16	Soil mesofauna diversity	Shannon index on subclass/suborder level	0.0–1.1	0.7 ± 0.3	Heat gradient extraction
17	Soil macrofauna diversity	Shannon index on family/order level	0.0–1.8	1.1 ± 0.4	Heat gradient extraction
18	Araneae diversity	Shannon index on species level	0.0–2.3	1.4 ± 0.6	Pitfall traps
19	Microbial respiration	µgCO_2_ − C/gsoil h	0.2–1.0	0.5 ± 0.2	CO_2_ evolution
20	Soil feeding activity	Average % of baits consumed	1.4–99.7	49.6 ± 30.7	Bait-lamina
21	Litter decomposition	Organic C/organic N (g)	54.1–128.8	80.2 ± 15.9	Litterbags
22	Soil water content	% water content/g dry soil	7.2–29.9	17.5 ± 6.1	Gravimetric
23	Soil mineral N	µg ammonium and nitrate/g dry soil	2.4–38.9	7.3 ± 7.1	Cd reduction and modified Berthelot reaction
24	C content wheat aboveground biomass	% C/g dry plant	0.8–3.4	2.0 ± 0.7	C/N analyses
25	N content wheat aboveground biomass	% N/g dry plant	42.0–45.9	43.8 ± 0.9	C/N analyses
26	Total aboveground wheat biomass	dry mass (t/ha)	2.1–22.4	10.1 ± 5.8	Subsampling and weighting

The total number of samples is N = 72, with the exception of variable 19 (N = 71) and variables 4, 5 and 21 (N = 70). For key references and detailed descriptions refer to the Suplementary information.

## Results

The sampling date (“Time”) and farming system (“Farming System”) explained the highest proportion of variation in the joint analysis of all data (Table 2; based on square-root transformed estimated components of variation), followed by the differences between the roof vs. both control treatments (Table 2, “Contrast”), the drought treatment (Table 2, ”Drought”), the interaction between sampling date and the roof vs. both control treatments (Table 2, “Time” × “Contrast”) and the interaction between sampling date and the drought treatment (Table 2, “Time” × “Drought”).

**Table 2:** Results of the permutational multivariate analysis of variance (PERMANOVA) of a resemblance matrix from Gower similarities between all pairs of 72 samples and the dependent variables 1–26 (Table 1).

Source	df	SS	MS	Pseudo-F	P(perm) or P(MC)*	Unique perms	Sq.root components of variation
Plot	6	2039.5	339.9	3.94	< 0.001	9903	5.31
Time	2	7951.9	3975.9	30.19	< 0.001	9934	12.66
Farming system (FS)	1	5856.6	5856.6	17.23	< 0.001*	35*	12.38
Drought	2	1084.6	542.3	4.97	< 0.0001	9941	4.25
Contrast	1	949.8	949.8	8.25	0.001	9954	5.11
Time × Drought	4	611.5	152.9	1.77	0.020	9906	2.89
Time × Contrast	2	499.3	249.7	2.81	< 0.001	9929	3.88
Time × FS × Drought	4	370.6	92.6	1.07	0.3925	9917	1.26
Time × FS × Contrast	2	207.9	103.9	1.17	0.3169	9948	1.68
Pooled (1)	14	1844.0	131.7	1.53	0.0179	9887	3.89
Pooled (2)	14	1528.1	109.2	1.27	0.1075	9866	2.76
Plot × Contrast	7	806.1	115.2	1.29	0.1065	9874	2.56
Residuals	24	2070.8	86.3				9.29
Total	71	23,358.0					

Pooled Estimated components of variation: (Pooled (1): Plot (nested in FS) × Time and Time × FS; Pooled (2): Plot (nested in FS) × Drought and FS × Drought).

*P-value derived from Monte-Carlo simulations due to small number (< 100) of unique permutations.

### Farming system and sampling date

**Figure 1:**
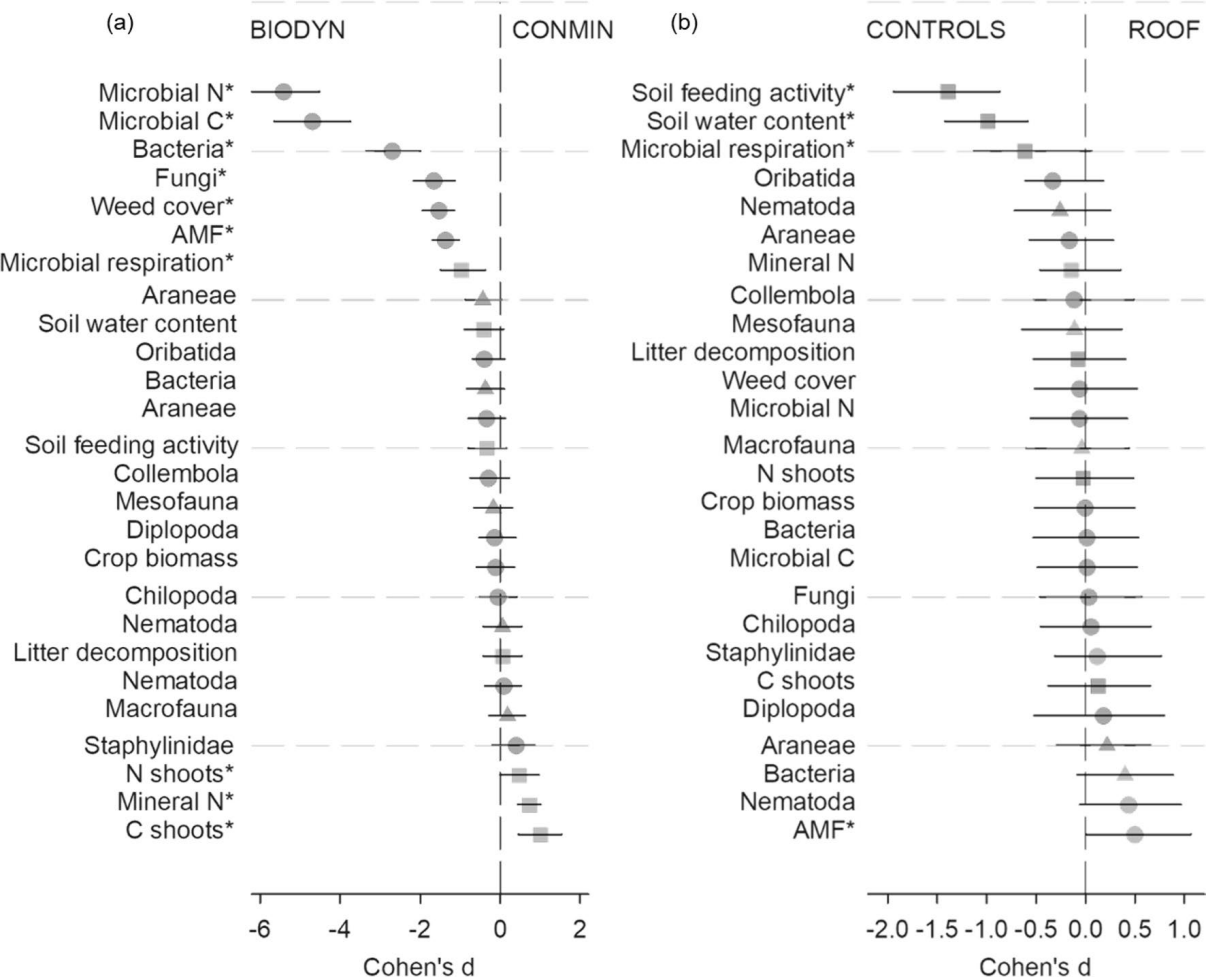
Effect size (Cohen’s d) for factors (**a**) “Farming System” and (**b**) drought “Contrast” (both controls vs. roof) on abundance- (●), diversity- (▲) and process-related (■) variables. The asterisk next to a variable name indicates a *P*-value < 0.05 that was derived from 5000 bootstrap samples.

The farming system affected microbial N and C, bacterial and fungal biomass, weed cover, AMF biomass and microbial respiration, all with significantly higher values in organic compared to conventional farming systems (Fig. 1a). The organic farming system had 1.9 times higher microbial N and 1.7 times higher microbial C values compared to the conventional farming system (Table s1).

**Figure 2:**
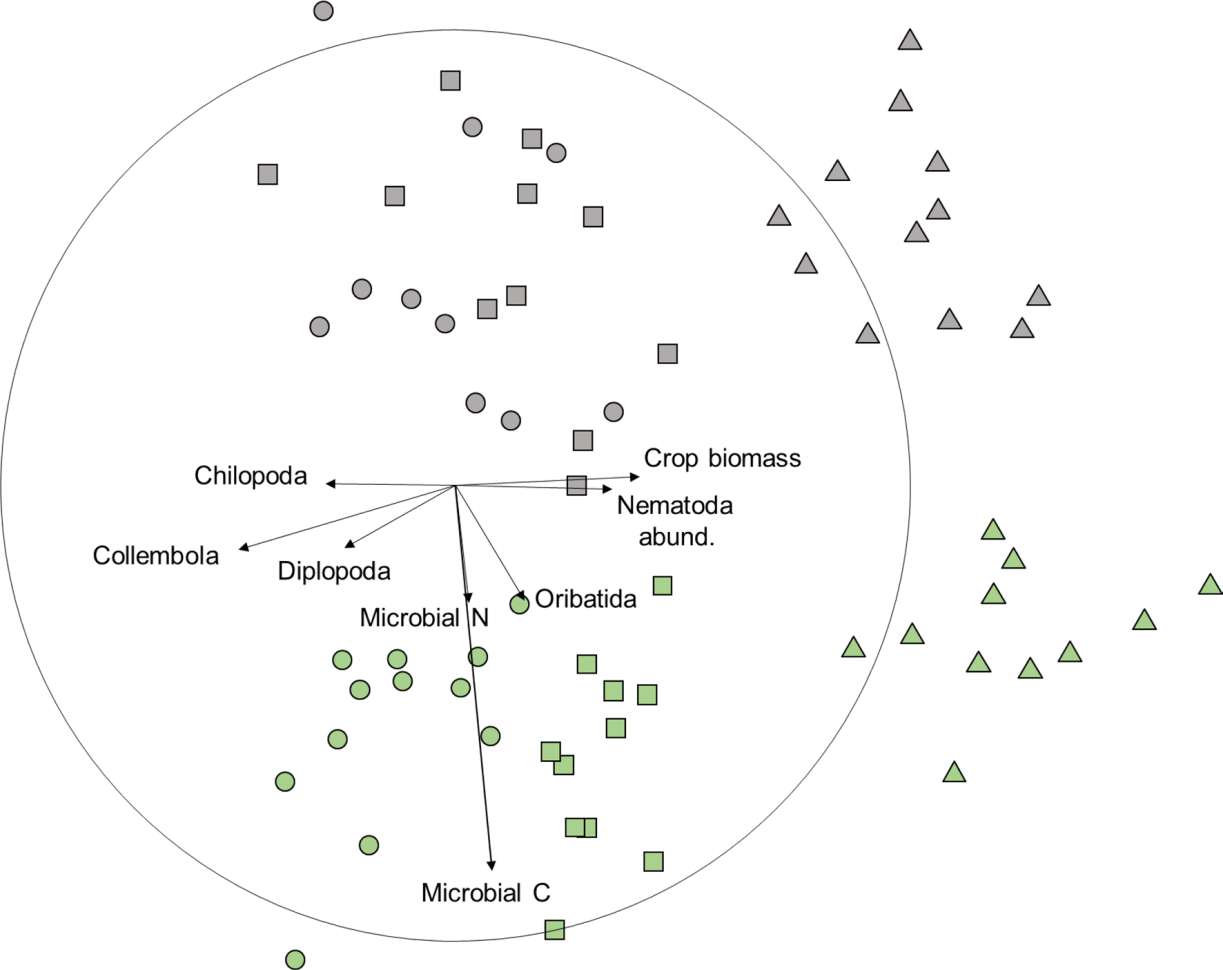
Non-metric multidimensional scaling (NMDS) ordination (2-d stress = 0.18) of farming systems (Green symbols organic, Grey symbols conventional) sampled at three dates (●T1, ■T2, ▲T3) based on a resemblance matrix from Gower distances of 26 abundance-, diversity- and process-related variables (see Table 1); vectors for individual variables were superimposed for variables with multiple correlation coefficients of R > 0.25, the circle indicates the highest possible multiple correlation coefficient.

The N content of aboveground crop biomass (1.2 times) and the mineral soil N content (2.0 times) was higher in the conventional farming system (Table s2). The magnitude of these effects was generally lower (than for those variables that had higher values in the organic farming system. The samples from the organic farming system clustered in the NMDS ordination in chronological order from left to right, whereas the samples from the conventional farming system were grouped into two clusters (sampling dates T1 and T2 vs. T3; Fig. 2).

Samples at sampling date T3 were on average characterized by a 1.5 and 1.7 times higher abundance of nematodes compared to T1 and T2 respectively (Table s1). Crop biomass was 3.7 and 2.2 times higher at T3 compared to T1 and T2. Samples at T1 had higher abundances of Chilopoda (4.0 times), Diplopoda (6.7 times) and Collembola (85.6 times) compared to T3 (Table s2).

### Experimental drought

Effects of the control and roof control treatment did not differ significantly in the joint analysis of all data (pairwise test; t = 1.15; *P* = 0.293). Follow-up analyses of drought effects therefore focused on the planned contrast between the roof (R) vs. both control treatments (C and RC). This focus is further supported as the importance of the “Contrast” model term was also higher than for the “Drought” term alone (Table 2: sq. root component of variation “Contrast” = 5.11 vs “Drought” = 4.25).

**Figure 3:**
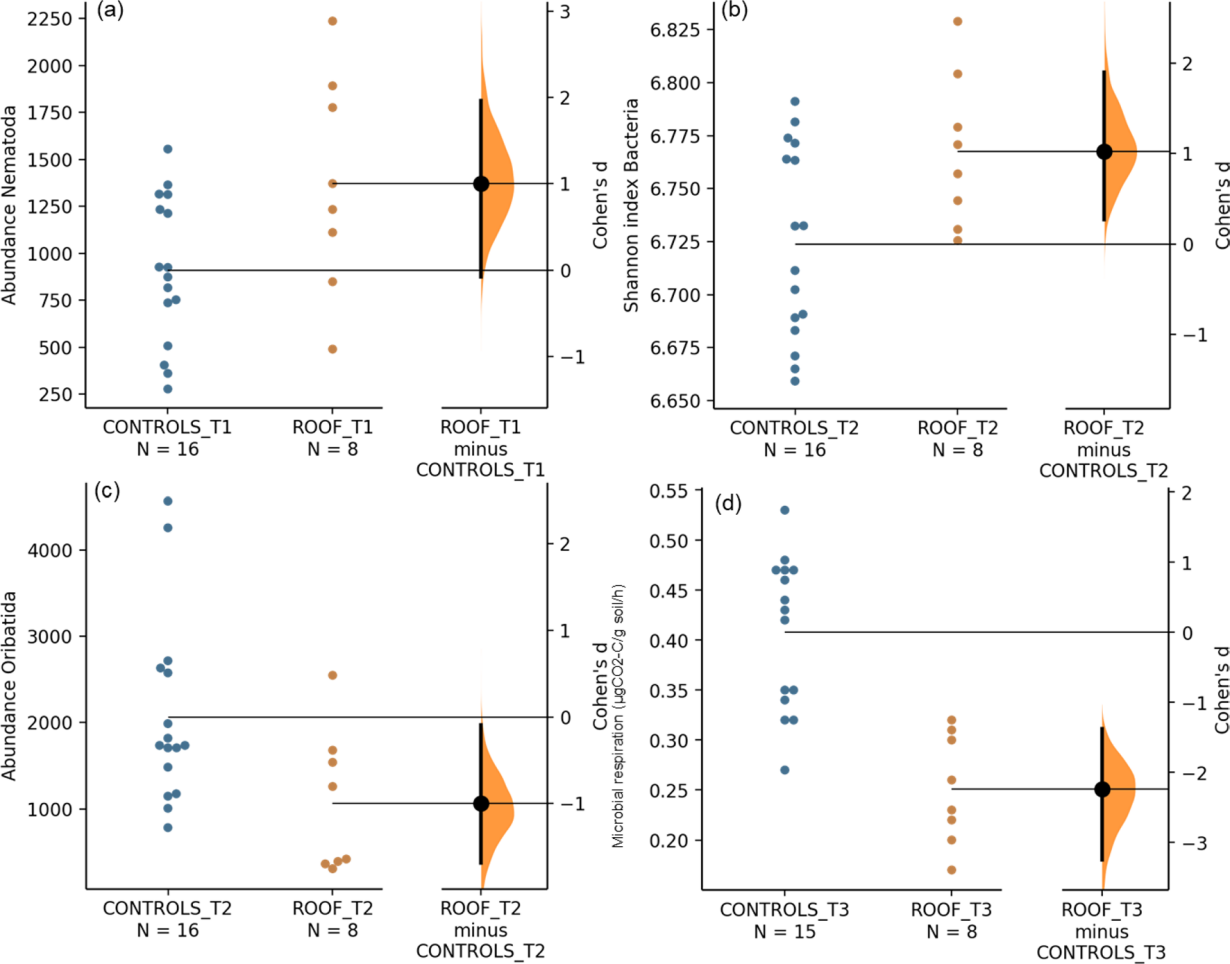
Effect size (Cohen's d) comparing Control (C & RC) vs. Roof (R) treatments at specific sampling dates (T1–T3) depicted as a black dot on the right axes for (**a**) Nematoda abundance at T1, (**b**) Bacterial diversity at T2, (**c**) Oribatida abundance at T2 and (**d**) Microbial respiration at T3. The distribution of Cohen's d is plotted on the axis on the right based on a bootstrap sampling distribution; the 95% confidence intervals are indicated by the ends of the vertical error bars. Raw data from each plot is shown in Control (Blue circle) and Roof (Orange circle) samples on the left axes.

The experimental drought significantly reduced the soil feeding activity (0.4 times), microbial respiration (0.8 times) and soil water content (0.7 times) and increased the biomass of AMF (1.4 times; Table s3) (Fig. 1b). The two most responsive variables, soil fauna feeding activity and soil water content, responded consistently and negatively to experimental drought conditions at all sampling dates. Four additional variables only responded to experimental drought at certain sampling dates. At T1, Nematoda abundance was higher under experimental drought when compared to the two controls (Fig. 3a) with a similar response pattern for bacterial diversity at T2 (Fig. 3b). In contrast, the abundance of Oribatida at T2 (Fig. 3c) and microbial respiration at T3 (Fig. 3d) were lowest under experimental drought.

### Relationships between diversity and functions

**Figure 4:**
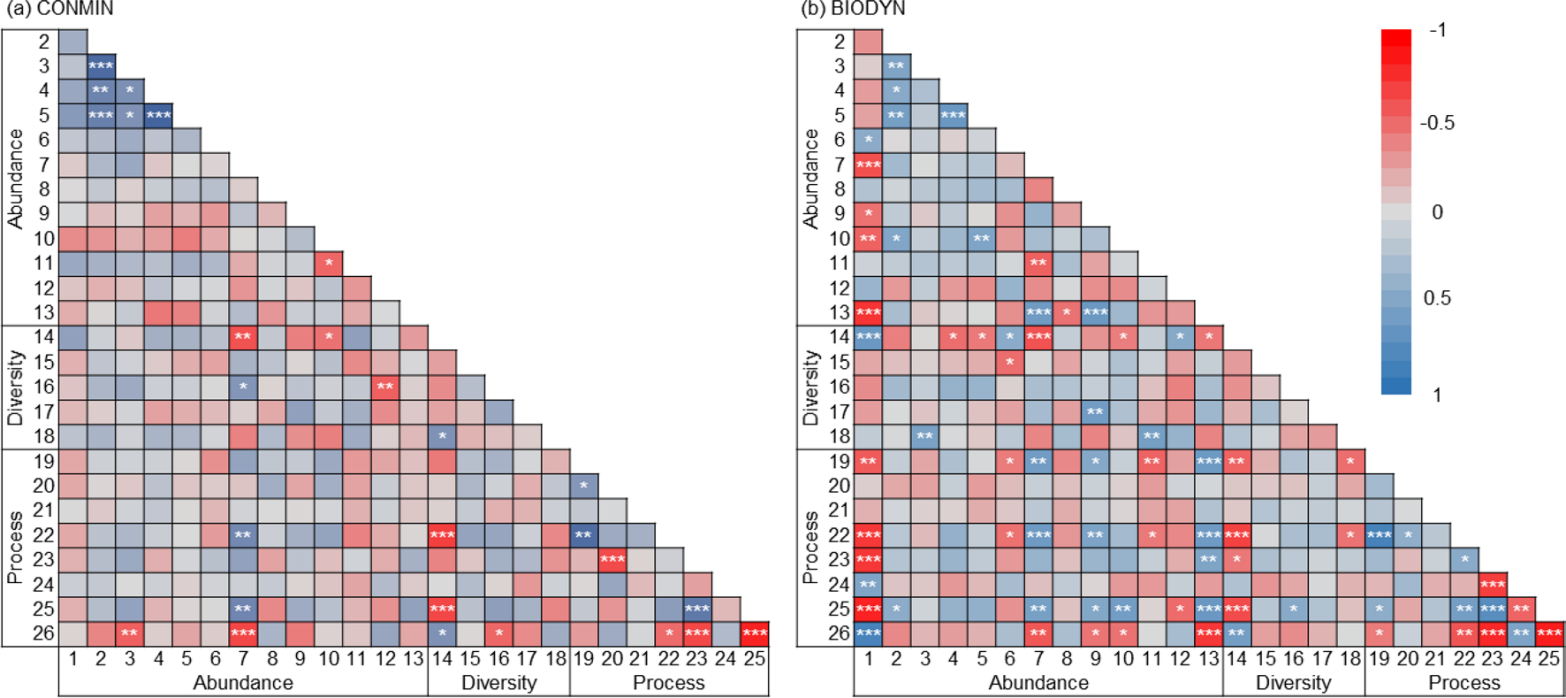
Spearman correlation matrices between all abundance, diversity and process-related dependent variables in the (**a**) conventional (N = 34–36) and (**b**) organic (N = 35–36) farming system. Cell colours indicate Spearman R-values according to the provided scale. Cells with asterisks indicate significant relationships after adjusting the *P*-values for multiple testing with to the Benjamini–Hochberg method and a False Discovery Rate of 0.05 (*P < 0.05, **P < 0.01, ***P < 0.001). Numbers of individual variables correspond to numbers in Table 1.

In the conventional farming system 14 pairs of dependent variables were significantly positively and 13 pairs were significantly negatively related (Fig. 4a). In the organic farming system, 38 pairs were significantly positively and 37 pairs were significantly negatively related (Fig. 4b). The overall number of significantly related variables was 2.8 times higher in the organic than in the conventional farming system (in total 75 vs. 27). A comparison of pairwise correlations between the roof and the control showed 18 positive and 7 negative significant correlations in the roof samples and 9 significant positive and 11 negative correlations in the control samples (25 vs. 20; Fig. s1a, b). The two correlation matrices for the roof and control treatments were more strongly related to each other (Mantel R = 0.76, lower 95% CI = 0.717, upper 95% CI = 0.792) than the two correlation matrices for the organic and conventional farming system (Mantel R = 0.59, lower 95% CI = 0.535, upper 95% CI = 0.661), as indicated by non-overlapping confidence intervals.

Variables associated with microbial abundance were positively related to each other across both farming systems and drought treatments, in particular bacterial (#2) and fungal biomass (#3), as well as microbial N (#4) and microbial C (#5). Significant positive correlations across the four matrices were further detected for soil water content (#22) and microbial respiration (#19). Crop biomass (#26) was significantly negatively related to mineral soil N content (#23) and N content of the crop aboveground biomass (#25) in all four correlation matrices. Bacterial diversity (#14) was consistently negatively related to soil water content (#22).

The 39 significant pairwise correlations between an abundance variable and a diversity- or process-related variable in the organic farming system (Fig. 4b) were only matched by 8 significant correlations in the resemblance matrix of the conventional farming system (Fig. 4a). Of this subset of correlations in particular, the 18 significant correlation pairs including AMF biomass (#1), Chilopoda abundance (#9) and weed cover (#13) were not observed in the conventional farming system. However, in both farming systems, Collembola abundance (#7) was related to soil water content (#22) and shoot N content (#25, both positive) and to bacterial diversity (#14) and crop biomass (#26, both negative). The number of significant relationships between pairs of abundance-related variables was also lower in the conventional than in the organic system (7 versus 15). The comparison between roof and control treatments did not show similar pronounced differences in pairwise correlations between factor levels (Fig. s1a, b). Collembola abundance, bacterial diversity, soil water content, shoot N content and aboveground crop biomass were likely strong drivers of the correlation patterns in both farming systems, as these variables were involved in a larger number of correlations (Fig. s2).

**Figure 5:**
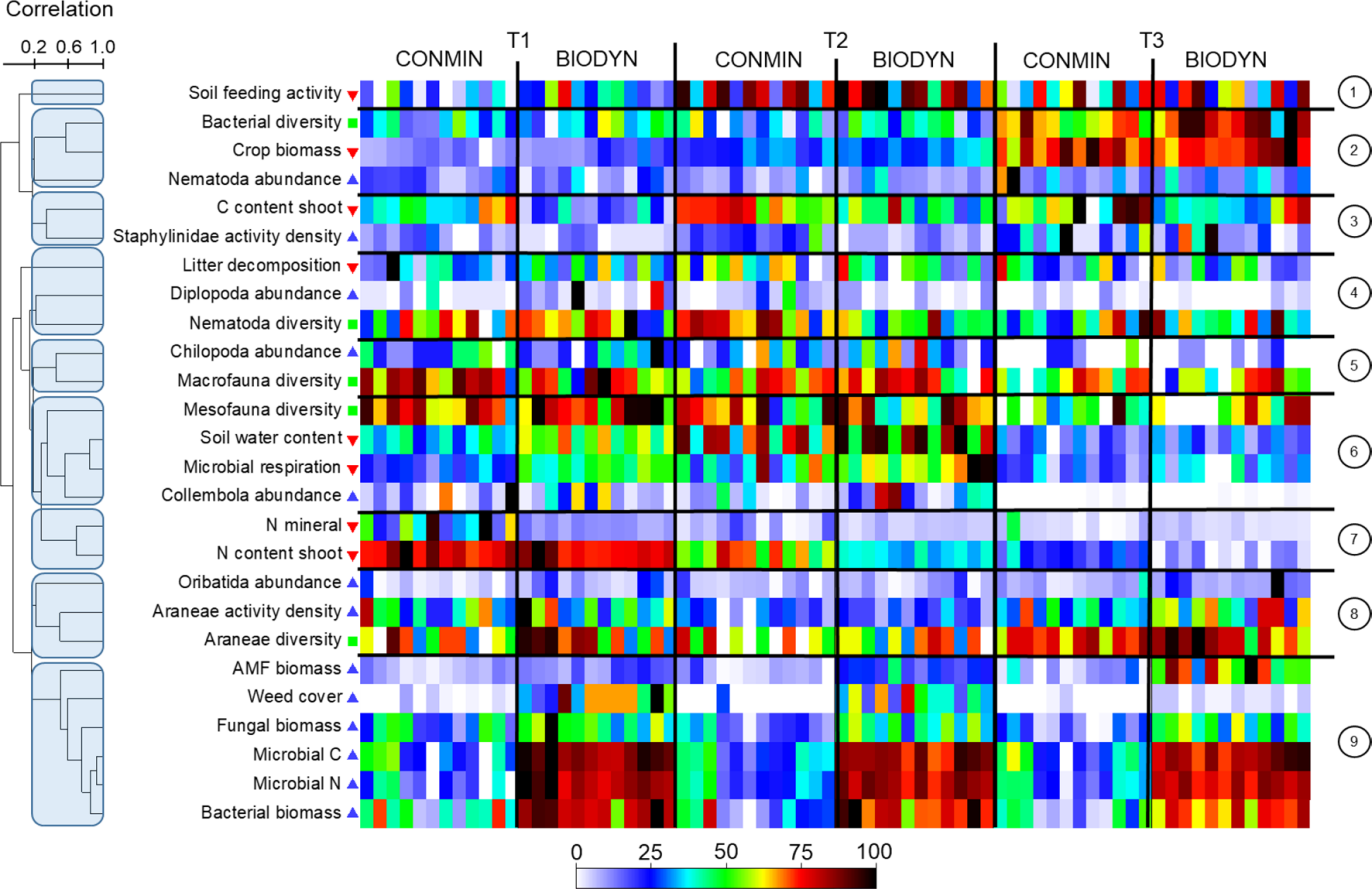
Based on pairwise correlations, the 26 analysed variables clustered into nine major bundles (numbers on the right and horizontal lines). The shade plot is based on individually standardized values for each dependent variable in Table 1 in all 72 samples (horizontal blocks) sorted by the factor “Farming System” (CONMIN, conventional vs. BIODYN, organic) within the factor “Time” (T1 to T3). The dendrogram and vertical order of the 26 dependent variables is based on a Spearman correlation matrix between all variables and a cluster analysis by group averaging (resulting clusters are highlighted by blue shading).

Based on pairwise correlations, the 26 analysed variables clustered into nine major bundles (1–9 in Fig. 5) primarily characterized by complex temporal patterns and comparable effects of the farming systems. Soil feeding activity, as the only variable in cluster 1, was consistently higher in the organic than in the conventional system and peaked at T2 after the highest precipitation during the study period. The three variables in cluster 2 peaked at T3 independent of the farming system, as for example, crop biomass was highest towards the end of the study. The two variables in cluster 3 had higher values in the conventional compared to the organic farming system across all sampling dates as for example Staphylinidae abundances were 1.6 times higher in the conventional system. Variables in cluster 4 had higher values in the organic than in the conventional system at T1, but showed the opposite pattern at T2. Variables in clusters 5 and 7 declined from T1 to T3, as for example the shoot N content. Variables in cluster 6, did not consistently change between T1 and T2, but were always lowest at T3 and on average higher in the organic than in the conventional system. Collembola, for example, almost disappeared at T3. Variables in cluster 8 had the lowest values at T2 at times of highest soil water content (see also cluster 1) and included abundance- and diversity-related variables (Araneae and Oribatida). Cluster 9 is composed exclusively of abundance-related variables which all had higher values in the organic than in the conventional farming system across all sampling dates.

## Discussion

It is important to understand the joint effects of land-use intensity and climate change on biodiversity and associated ecosystem functions in arable soils. Addressing this knowledge gap will facilitate the development of sustainable approaches for future agricultural management in a rapidly changing world^3^. The conventional farming system and experimental drought affected several soil variables negatively. We did not observe strong additive or interactive effects of the two global change aspects on soil organisms or functions (only partly supporting our first hypothesis). The significant response of soil processes (3 out of 8 processes), rather than abundance or diversity variables (1 out of 18 variables), to short-term experimental drought suggests that activity measures may be suitable early warning indicators for drought stress in arable soils.

The almost three times higher number of observed significant correlations between pairs of soil variables in the organic farming system, suggests a considerably weaker linkage between soil biota and soil processes under long-term conventional farming (supporting our third hypothesis). A comparable loss of relationships between biodiversity and ecosystem functions with increasing land-use intensity has been documented in grassland and forest ecosystems^36^. Our study, for the first time, shows that a comparable loss also occurs under conventional agriculture, but not due to experimental drought conditions. The observed high number of significant relationships between soil variables under organic agriculture and a higher soil water content during early and intermediate stages of the growing season suggest that organic farming may indeed provide a buffer against drought effects (supporting our second hypothesis).

### Farming systems and sampling date

The sampling date affected soil organisms and functions, with pronounced differences in crop biomass and soil water content between dates potentially acting as major drivers of other variables. Crop biomass was highest and soil water content was below the estimated wilting point of approximately 14% at the latest sampling date. At this sampling date, soil water content reached an average of 10.3% of dry weight accompanied by the highest nematode, but lowest soil macro- (Myriapoda) and micro-arthropod (Collembola) abundance (see also^46^). Given the limitations of complex field experiments, it is not possible to conclude on the major mechanistic links between variables that could cause the observed relationships. Soil water content was, however, higher in the organic farming system at T1 and T2 compared to the conventional system^38^. Soil organisms such as Collembola or soil functions such as microbial respiration were positively related to soil water content. Collembola are sensitive to drought conditions and may actively move into deeper soil layers to avoid unfavourable conditions and desiccation^47,48^. It is notable, that not only the abundance, but also the diversity of soil animals (meso- and macrofauna) was lowest at the latest sampling date when the soil water content was lowest, a pattern previously observed for Collembola^18^. This pattern was mirrored by soil feeding activity reaching its peak values when soil water content, microbial biomass and respiration were highest. Our results suggest that soil water content plays a vital role for the abundance of soil mesofauna and feeding activity in temperate agroecosystems.

Bacteria, fungi and arable weeds consistently had higher abundances in the organically managed plots, which confirms previous results from the DOK trial^38,49,50^ and wheat fields in general (for a review see^51^). In contrast, the C and N content of crop shoots and soil mineral N content were higher in the conventional farming system. This pattern likely results from the application of synthetic NPK fertilizer in the conventional, but not the organic farming system. Rove beetles (Coleoptera, Staphylinidae) were more abundant in the conventionally managed plots. Previous studies documented higher densities of cereal aphids in conventionally managed wheat plots^49^ and suggested that some rove beetle species show a strong preference for aphid prey in the DOK trial^52^.

### Experimental drought

Similar to the observed sensitivity of Collembola and soil fauna feeding activity to ambient seasonal drought conditions, short-term experimental drought also significantly reduced soil fauna feeding activity^53^. Ambient drought conditions over the growing season may have reduced the abundance of detritivorous microarthropods below a threshold under which additional effects of experimental short term drought did not reduce feeding activity any further.

Microbial respiration, was jointly reduced by ambient drought conditions over the growing season and by the experimental drought treatment. The almost complete loss of major soil fauna groups at times of severe ambient (Collembola, Chilopoda, Diplopoda) or experimental (Oribatida) drought conditions, and the pronounced response of microbial activity to drought is alarming considering predictions for future climatic conditions in central Europe. Notably, only very few soil organisms responded positively either to experimental (Nematoda at T1) or ambient and experimental drought conditions (AMF). The observed higher AMF abundance under experimental drought conditions might be driven by increased carbon allocation from the plant to the symbiotic fungus in times of drought. The observed contrasting patterns of different soil organisms highlight the complex responses that need to be considered to predict changes in soil food webs under future climate change^54^.

### Relationships between diversity and functions

In previous experiments, altered precipitation modified relationships between above- and below-ground biota, inducing a “climatic decoupling”^55^. The number of significant relationships between soil biota and process-related variables was almost three times higher in the organic than in the conventional farming system. Contrary to our hypothesis and previous results, experimental short-term drought did not result in fewer significant relationships compared to the control treatment. These results suggest a considerable weakening of pairwise relationships due to long-term conventional farming, but not due to experimental drought conditions.

A particularly strong decline in the number of significant pairwise relationships in response to long-term conventional agriculture was observed for AMF biomass and 12 other variables. On average, AMF biomass was more than twice as high in the organic than in the conventional farming system. The observed loss of multiple significant relationships suggests that AMF biomass dropped below a critical threshold value under long-term conventional management. In the organic farming system, AMF biomass was higher in plots with lower soil arthropod abundance (Chilopoda, Diplopoda and Collembola). Collembola and Diplopoda are known to incorporate AMF into their diet either by feeding on roots or indirectly by consumption of dead organic matter^56,57^. Collembola may even suppress AMF hyphal networks in agricultural soils to an extent to which nitrogen uptake by crop plants is reduced^58^. Our results, however, suggest that Collembola abundance was positively related to N content of wheat shoots in both farming systems. This result indicates beneficial effects of Collembola for plant growth irrespective of potential negative effects on AMF biomass.

AMF biomass was negatively correlated to several process-related variables in organically managed plots including microbial respiration, mineral N content of soil and N content of wheat shoots. It is important to consider that another variable, soil water content, was also negatively related to AMF biomass and was positively related (at least under organic agriculture) to mineral N content of soil and N content of wheat shoots. This example highlights that two processes may be correlated due to (i) a direct (causal) relationship, (ii) correlated drivers or may share (iii) an indirect relationship due to shared, but uncorrelated, drivers (Fig. 1 in^33^). In this study, relationships between AMF biomass and process-related variables may at least partly stem from indirect relationships mediated via a shared driver (soil water content). However, the crucial role of AMF in nitrogen cycles of agricultural soils suggests that at least some relationships to the N content of soil or crop plants result from direct relationships^42,59^.

Nine positive relationships between arthropod or weed abundance and process-related variables were significant in organically, but not in conventionally managed plots. As with AMF biomass, these relationships may partly stem from a shared driver, as arthropod and plant abundances as well as process-related variables were all also related to soil water content in the organic farming system.

Soil cover with arable weeds was very low in the conventional (1.4%) compared to the organic system (29.9%) suggesting that weed cover directly affected several process-related variables in the organic farming system. The positive correlation between weed cover and soil water content in the organic but not in the conventional farming system suggests that weed cover reduced evaporation. Wheat biomass was significantly positively related to microbial diversity in both farming systems. Carson et al.^60^ showed that drought reduces the connectivity of soil pores creating isolated habitats that facilitates the establishment of less competitive bacteria. Drought-induced shifts in pore connectivity may therefore promote bacterial diversity, as it was also highest at the driest sampling date in this study.

## Conclusions

The observed complex results for soil biota and associated processes can only partly be explained by this study due to general limitations of complex field experiments. Our results on the reorganization of relationships nevertheless highlight the importance to jointly consider global change drivers under realistic field conditions. While activity metrics are suitable to indicate short-term drought stress, community metrics seem to be particularly sensitive to seasonal changes in drought conditions, and in the case of microbial communities to agricultural practices. The loss of multiple pairwise relationships between soil biota and process-related variables due to conventional agriculture indicates shifts in ecosystem functioning in response to long-term farming practices. This result implies that soil ecosystem functions as fundamental components of ecological intensification approaches may be threatened under long-term conventional agriculture. The higher soil water content during early and intermediate stages of the growing season and the high number of significant relationships under organic agriculture support the hypothesis that organic farming may provide a buffer against drought effects.

## Material and methods

### Study site

The study was performed in 2017 in the DOK trial (bioDynamic, bioOrganic, Konventionell [Conventional]), a Swiss agricultural long-term farming system comparison (47° 30′ 09.3′′ N, 7° 32′ 21.5′′ E, 300 m above sea level) established in 1978^50^. Different organic and conventional farming systems were established on replicated on a Haplic Luvisol soil on deep sediments of loess^61^; over the previous five years, the mean annual temperature at the site was 10.5 °C and the mean annual precipitation 890 mm^30^. The study was performed in plots of winter wheat (*Triticum aestivum* L. cv. ‘Wiwa’) in an organic (biodynamic, BIODYN) and conventional (CONMIN) farming system (factor “Farming System”) with soybeans as preceding crop. Plots in both farming systems are ploughed to 20 cm depth and are managed according to the same 7-year crop rotation. The organic farming system has been managed according to the guidelines for ‘Demeter’ food production (https://demeter.ch/) for the last 40 years and thus relies exclusively on organic fertilisation (slurry, composted animal manure), biological pest and mechanical weed control and applies biodynamic preparations to soils, plant and compost^30^. The conventional farming system has only received (synthetic) mineral fertilizer according to Swiss guidelines^62^ in addition to insecticide, herbicide and fungicide applied according to threshold values and application doses as recommended by the producer. Over the first 35 years, the average level of applied total N was 17% lower, but the directly effective ammonia and nitrate level were 75% lower in BIODYN as compared to CONMIN^61^. Further details on management operations conducted during the experimental phase are listed in^38^.

### Experimental design

Natural precipitation levels were manipulated (Factor “Drought”) in each of the four replicated plots (5 m × 20 m) per farming system, by setting up passive, fixed-location, partial rainout shelters (2.5 m × 2.5 m, 1.3–1.7 m height) along with two different control treatments, resulting in 24 experimental subplots (four replicated plots in two farming systems with three drought treatment subplots each). The three drought subplots were (a) a partial rainout shelter reducing precipitation by 65% (Roof, R), (b) a rainout shelter control (Roof Control, RC), which did not actively reduce precipitation, but mimics potential microclimatic artefacts of the rainout shelter and (c) an open field control without a shelter (Control, C). For details on the design of the rainout shelter and experimental set-up see Kundel et al.^38,63^. The shelters were set up in mid-March at the tillering stage of wheat and removed in June 2017, shortly before the final wheat harvest in the DOK trial.

### Sampling

Table 1 provides an overview of the sampled soil biota groups (#1–18) and process-related variables (#19–26). Following definitions in Garland et al.^64^ we grouped process-related variables as indicators of ecosystem functions, defined as “biotic or abiotic processes that occur within an ecosystem and may contribute to ecosystem services either directly or indirectly”. All variables were sampled at three different sampling dates (Factor “Time”) during the wheat growing season in mid-April (T1, 4 weeks after the establishment of subplots, wheat Zadok stage 31–32), mid-May (T2, 8 weeks, Zadok stage 38–39) and mid-June (T3, 13 weeks, Zadok stage 75), respectively. An overview of sampling methods and approaches for individual variables is listed in Table 1, details are provided in the Supplementary information.

### Statistical analyses

Permutational multivariate analysis of variance (PERMANOVA^65^) was used to jointly analyse responses 1–26 as dependent variables (Table 1). The 26 dependent variables were transformed into a single resemblance matrix with pairwise distances between all 72 samples based on Gower similarities. By using Gower similarities, all variables were internally standardized to range from 0–1 and data was then transformed into a resemblance matrix with percentage similarity values for all pairs of samples. The random factor “Plot” (8 levels, nested in farming system) and the fixed factors “Farming System” (2 levels: CONMIN, & BIODYN) and “Drought” (3 levels: R, RC& C) and “Time” (3 levels: T1 to T3) were specified for the PERMANOVA model. In addition, the planned “Contrast”: R vs. both control treatments (C + RC) was specified. PERMANOVA was then performed with type III sums of squares and 9999 permutation of residuals under a reduced model. In case of low number of unique permutations for the fixed factor “Farming System”, P-values were calculated from Monte-Carlo simulations^65^.

The resulting estimated components of variation were used to compare the relative importance of different model terms towards explaining the overall variation in the multivariate data. Components with negative estimates were removed from the model by pooling, starting with the component with the most negative mean square (MS) value. Pooling continued until only components with positive estimates were left as recommended^66^. Follow-up analyses for significant model terms were then performed according to the following procedures.

To identify the magnitude of the main effects (factors “Farming System” and “Drought”) Cohen’s d was calculated as a measure of effect size for individual variables and results for all variables are shown as forest plots. To analyse the interaction term between factor “Time” and the planned “Contrast”, Cohen’s d was calculated for each variable at each sampling date and shown as estimation plots^67^. To visualize the effects of the factor “Time” on individual dependent variables, non-metric multidimensional scaling ordination (NMDS) was constructed based on the Gower resemblance matrix and the Kruskal stress formula 1 with 500 restarts, and a minimum stress of 0.001. Vectors for individual variables were then superimposed on the two-dimensional NMDS ordination for all variables with multiple correlation coefficients of R > 0.25. Note that 5 out of 72 samples had to be omitted for the NMDS ordination due to missing values for single dependent variables (see Table 1 for more details).

To analyse how differences between levels of the factors “Farming System” or “Drought” affected pairwise relationships between dependent variables, correlation matrices based on the Spearman correlation coefficient were calculated for individual factor levels. All P-values in these matrices were then adjusted for multiple testing using the Benjamini–Hochberg method with a False Discovery Rate of 0.05. To identify bundles of variables that are related to each other and shared comparable response patterns to the factors “Time” and “Drought” a shade plot was created based on individually standardized values for each dependent variable (standardization by maximum, ranging from 0–100 for each variable). These values were then plotted for each dependent variable according to a colour gradient with samples sorted horizontally by factor “Time” within the two levels of factor “Farming System”. Dependent variables were then sorted vertically according to a Spearman correlation matrix of all pairwise relationships between dependent variables and a resulting cluster analysis based on group averaging. PERMANOVA models, ordinations, cluster analysis and the shade plot were calculated with PRIMER 7 version 7.0.13 and the PERMANOVA add-on (PRIMER-e, Quest Research Limited, Auckland, New Zealand). Cohen’s d and Gardner-Altman estimation plots were calculated according to^67^ on https://www.estimationstats.com/. Mantel tests and confidence intervals were calculated in R version 4.1.0^68^ using the R package phytools^69^.

## Supplementary Information

Supplementary Information.

## Data Availability

Data is available at 10.6084/m9.figshare.17125169.

## References

[1] Baer SG, Birgé HE (2018). Soil ecosystem services: An overview. Manag. Soil Health Sustain. Agric..

[2] Geisen S, Wall DH, van der Putten WH (2019). Challenges and opportunities for soil biodiversity in the anthropocene. Curr. Biol..

[3] Guerra CA (2021). Tracking, targeting, and conserving soil biodiversity. Science.

[4] Tsiafouli MA (2015). Intensive agriculture reduces soil biodiversity across Europe. Global Change Biol..

[5] Bender SF, Wagg C, van der Heijden MGA (2016). An underground revolution: Biodiversity and soil ecological engineering for agricultural sustainability. Trends Ecol. Evol..

[6] Wagg C, Bender SF, Widmer F, van der Heijden MGA (2014). Soil biodiversity and soil community composition determine ecosystem multifunctionality. PNAS.

[7] Wall DH, Nielsen UN, Six J (2015). Soil biodiversity and human health. Nature.

[8] Smith P (2016). Global change pressures on soils from land use and management. Global Change Biol..

[9] Birkhofer, K., Smith, H. G. & Rundlöf, M. Environmental Impacts of Organic Farming. in *eLS*. 1–7 (John Wiley & Sons Ltd, 2016).

[10] Bengtsson J, Ahnström J, Weibull A-C (2005). The effects of organic agriculture on biodiversity and abundance: A meta-analysis: Organic agriculture, biodiversity and abundance. J. Appl. Ecol..

[11] Abbott LK, Manning DAC (2015). Soil health and related ecosystem services in organic agriculture. Sustain. Agric. Res..

[12] de Graaff, M.-A., Hornslein, N., Throop, H. L., Kardol, P. & van Diepen, L. T. A. Effects of agricultural intensification on soil biodiversity and implications for ecosystem functioning: A meta-analysis. in *Advances in Agronomy* vol. 155 1–44 (Elsevier, 2019).

[13] Peters MK (2019). Climate–land-use interactions shape tropical mountain biodiversity and ecosystem functions. Nature.

[14] Pokhrel Y (2021). Global terrestrial water storage and drought severity under climate change. Nat. Clim. Change.

[15] Iglesias A, Garrote L (2015). Adaptation strategies for agricultural water management under climate change in Europe. Agric. Water Manage..

[16] Pörtner, H. O. *et al. IPBES-IPCC Co-sponsored Workshop Report Synopsis on Biodiversity and Climate Change*. https://zenodo.org/record/4920414 (2021).

[17] Blankinship JC, Niklaus PA, Hungate BA (2011). A meta-analysis of responses of soil biota to global change. Oecologia.

[18] Holmstrup M (2017). Long-term and realistic global change manipulations had low impact on diversity of soil biota in temperate heathland. Sci. Rep..

[19] Fry EL (2018). Soil multifunctionality and drought resistance are determined by plant structural traits in restoring grassland. Ecology.

[20] Zhou Z, Wang C, Luo Y (2020). Meta-analysis of the impacts of global change factors on soil microbial diversity and functionality. Nat. Commun..

[21] Schimel JP (2018). Life in dry soils: Effects of drought on soil microbial communities and processes. Annu. Rev. Ecol. Evol. Syst..

[22] Kundel D (2021). Drought effects on nitrogen provisioning in different agricultural systems: Insights gained and lessons learned from a field experiment. Nitrogen.

[23] Abbasi AO (2020). Reviews and syntheses: Soil responses to manipulated precipitation changes: An assessment of meta-analyses. Biogeosciences.

[24] Webber H (2018). Diverging importance of drought stress for maize and winter wheat in Europe. Nat. Commun..

[25] Gomez-Zavaglia A, Mejuto JC, Simal-Gandara J (2020). Mitigation of emerging implications of climate change on food production systems. Food Res. Int..

[26] Yin R (2020). Soil functional biodiversity and biological quality under threat: Intensive land use outweighs climate change. Soil Biol. Biochem..

[27] Rawls WJ, Pachepsky YA, Ritchie JC, Sobecki TM, Bloodworth H (2003). Effect of soil organic carbon on soil water retention. Geoderma.

[28] Lal R (2016). Soil health and carbon management. Food Energy Secur..

[29] Iizumi T, Wagai R (2019). Leveraging drought risk reduction for sustainable food, soil and climate via soil organic carbon sequestration. Sci. Rep..

[30] Fließbach A, Oberholzer H-R, Gunst L, Mäder P (2007). Soil organic matter and biological soil quality indicators after 21 years of organic and conventional farming. Agric. Ecosyst. Environ..

[31] Gattinger A (2012). Enhanced top soil carbon stocks under organic farming. PNAS.

[32] Schädler M (2019). Investigating the consequences of climate change under different land-use regimes: A novel experimental infrastructure. Ecosphere.

[33] Birkhofer K (2015). Ecosystem services: Current challenges and opportunities for ecological research. Front. Ecol. Evol..

[34] Birkhofer K (2018). Relationships between multiple biodiversity components and ecosystem services along a landscape complexity gradient. Biol. Cons..

[35] Chabert A, Sarthou J-P (2020). Conservation agriculture as a promising trade-off between conventional and organic agriculture in bundling ecosystem services. Agric. Ecosyst. Environ..

[36] Felipe-Lucia MR (2020). Land-use intensity alters networks between biodiversity, ecosystem functions, and services. PNAS.

[37] Lori M, Symnaczik S, Mäder P, De Deyn G, Gattinger A (2017). Organic farming enhances soil microbial abundance and activity: A meta-analysis and meta-regression. PLoS ONE.

[38] Kundel D (2020). Effects of simulated drought on biological soil quality, microbial diversity and yields under long-term conventional and organic agriculture. FEMS Microbiol. Ecol..

[39] Chen Q-L (2020). Rare microbial taxa as the major drivers of ecosystem multifunctionality in long-term fertilized soils. Soil Biol. Biochem..

[40] Garland G (2021). Crop cover is more important than rotational diversity for soil multifunctionality and cereal yields in European cropping systems. Nat. Food.

[41] Tamburini G (2020). Agricultural diversification promotes multiple ecosystem services without compromising yield. Sci. Adv..

[42] Vazquez C, de Goede RGM, Rutgers M, de Koeijer TJ, Creamer RE (2020). Assessing multifunctionality of agricultural soils: Reducing the biodiversity trade-off. Eur. J. Soil. Sci..

[43] Zwetsloot MJ (2020). Soil multifunctionality: Synergies and trade-offs across European climatic zones and land uses. Eur. J. Soil. Sci..

[44] Delgado-Baquerizo M (2017). Soil microbial communities drive the resistance of ecosystem multifunctionality to global change in drylands across the globe. Ecol. Lett..

[45] Bardgett RD, Caruso T (2020). Soil microbial community responses to climate extremes: Resistance, resilience and transitions to alternative states. Phil. Trans. R. Soc. B.

[46] Meyer S, Kundel D, Birkhofer K, Fliessbach A, Scheu S (2021). Soil microarthropods respond differently to simulated drought in organic and conventional farming systems. Ecol. Evol..

[47] De Smedt P (2018). Linking macrodetritivore distribution to desiccation resistance in small forest fragments embedded in agricultural landscapes in Europe. Landscape Ecol..

[48] Liu WPA, Phillips LM, Terblanche JS, Janion-Scheepers C, Chown SL (2021). An unusually diverse genus of Collembola in the Cape Floristic Region characterised by substantial desiccation tolerance. Oecologia.

[49] Birkhofer K (2008). Long-term organic farming fosters below and aboveground biota: Implications for soil quality, biological control and productivity. Soil Biol. Biochem..

[50] Mäder P (2002). Soil fertility and biodiversity in organic farming. Science.

[51] Birkhofer, K., Bezemer, T. M., Hedlund, K. & Setälä, H. Community composition of soil organisms under different wheat farming systems. in *Microbial Ecology in Sustainable Agroecosystems* 89–111 (CRC press Boca Raton, 2012).

[52] Birkhofer K (2011). Soil fauna feeding activity in temperate grassland soils increases with legume and grass species richness. Soil Biol. Biochem..

[53] Siebert J (2019). Extensive grassland-use sustains high levels of soil biological activity, but does not alleviate detrimental climate change effects. Adv. Ecol. Res..

[54] de Vries FT (2012). Land use alters the resistance and resilience of soil food webs to drought. Nat. Clim. Change.

[55] Torode MD (2016). Altered precipitation impacts on above-and below-ground grassland invertebrates: Summer drought leads to outbreaks in spring. Front. Plant Sci..

[56] Jonas JL, Wilson GWT, White PM, Joern A (2007). Consumption of mycorrhizal and saprophytic fungi by Collembola in grassland soils. Soil Biol. Biochem..

[57] Susanti WI, Pollierer MM, Widyastuti R, Scheu S, Potapov A (2019). Conversion of rainforest to oil palm and rubber plantations alters energy channels in soil food webs. Ecol. Evol..

[58] Seres A (2009). Collembola decrease the nitrogen uptake of maize through arbuscular mycorrhiza. ekol.

[59] Bender SF, van der Heijden MGA (2015). Soil biota enhance agricultural sustainability by improving crop yield, nutrient uptake and reducing nitrogen leaching losses. J. Appl. Ecol..

[60] Carson JK (2010). Low pore connectivity increases bacterial diversity in soil. Appl. Environ. Microbiol..

[61] Krause, H.-M., Fliessbach, A., Mayer, J. & Mäder, P. Implementation and management of the DOK long-term system comparison trial. in *Long-Term Farming Systems Research* 37–51, (Elsevier, 2020).

[62] Richner W (2017). Grundlagen für die Düngung landwirtschaftlicher Kulturen in der Schweiz (GRUD 2017). Agrarforschung Schweiz.

[63] Kundel D (2018). Design and manual to construct rainout-shelters for climate change experiments in agroecosystems. Front. Environ. Sci..

[64] Garland G (2021). A closer look at the functions behind ecosystem multifunctionality: A review. J. Ecol..

[65] Anderson, M. J. Permutational Multivariate Analysis of Variance (PERMANOVA). in *Wiley StatsRef: Statistics Reference Online* 1–15.

[66] Fletcher DJ, Underwood AJ (2002). How to cope with negative estimates of components of variance in ecological field studies. J. Exp. Mar. Biol. Ecol..

[67] Ho J, Tumkaya T, Aryal S, Choi H, Claridge-Chang A (2019). Moving beyond P values: data analysis with estimation graphics. Nat. Methods.

[68] R Core Team. R: A Language and Environment for Statistical Computing. R Foundation for Statistical Computing. Vienna, Austria. https://www.R-project.org.

[69] Revell LJ (2012). phytools: An R package for phylogenetic comparative biology (and other things). Methods Ecol. Evol..

