# Supplementary material for: Conventional agriculture and not drought alters relationships between soil biota and functions
Source: Sci Rep. 2021 Dec 14;11:23975. doi: 10.1038/s41598-021-03276-x (PMC8671559; doi:10.1038/s41598-021-03276-x)
Supplement: Supplementary file 1 — Supplementary Information. [file 41598_2021_3276_MOESM1_ESM.docx]

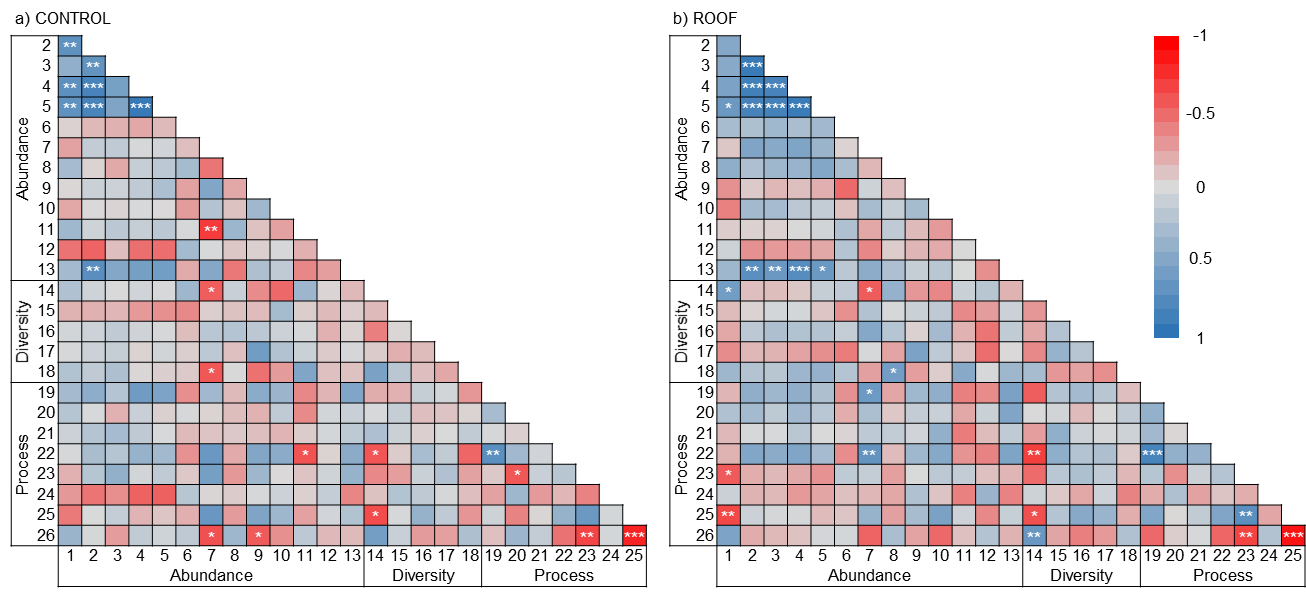


**Supplementary Figure s1** Spearman correlation matrix between all abundance, diversity and process-related variables in plots of the a) CONTROL (N=23-24) or b) ROOF (N=22-24) farming system. Cell colours indicate Spearman R-values according to the provided scale. Cells with asterisks are significant after adjusting the P-value for multiple testing using the Benjamini-Hochberg method with a false Discovery Rate of 0.05 (* P<0.05, ** P<0.01, *** P<0.001). Numbers of individual variables correspond to numbers in Tab. 2.


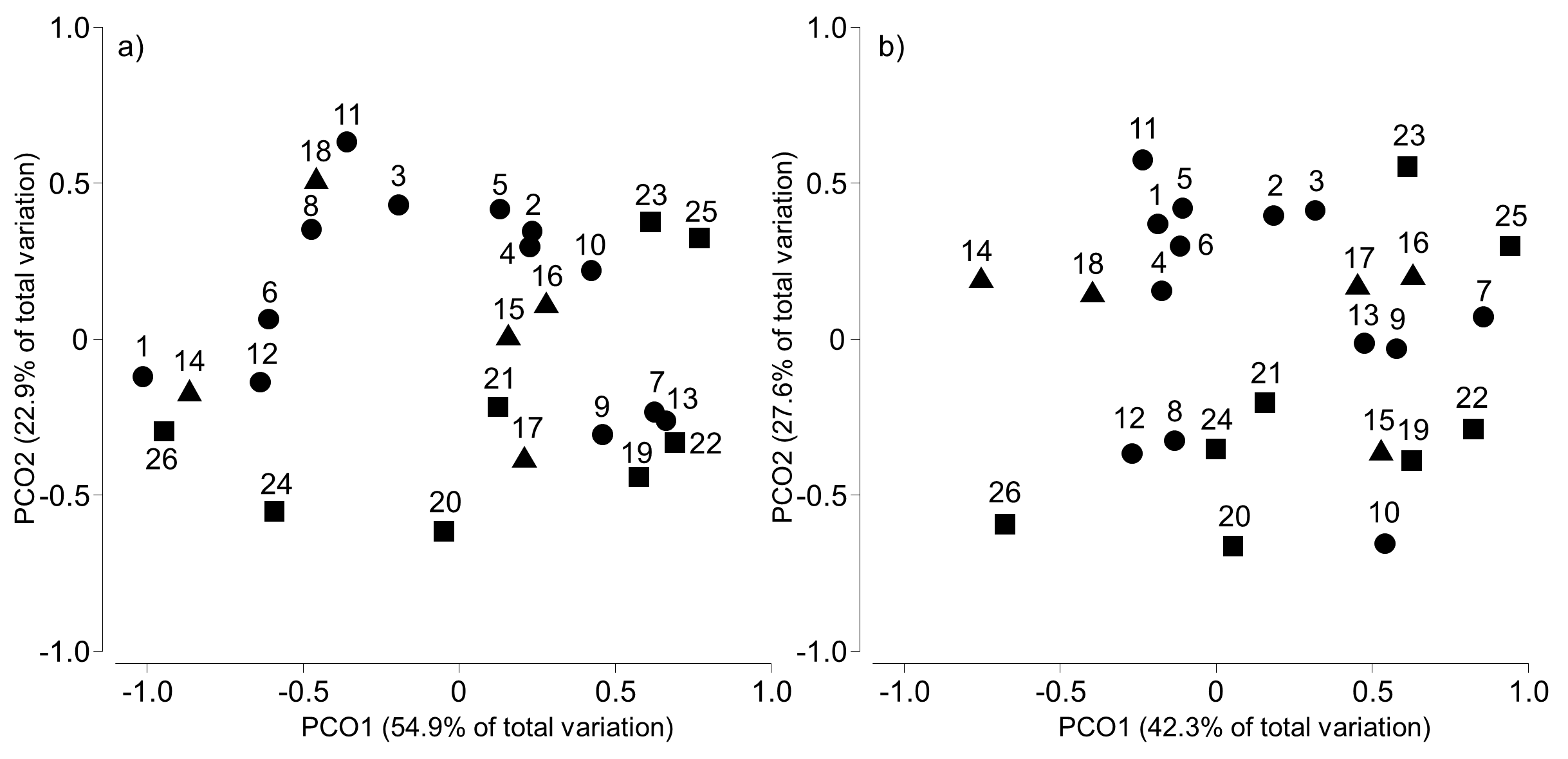


**Supplementary Figure s2** Principle coordinates analysis ordination (PCO) based on the Spearman R correlation coefficients between pairs of abundance- (●), diversity- (▲) and process-related (■) dependent variables (1-26 see variables names in Tab. 2) in the a) organic (BIODYN) and b) conventional (CONMIN) farming system**Supplementary Table s1** Means and standard deviation of individual factor levels of fixed factor “Farming System” and all 26 dependent variables.

|  |  | CONMIN | | BIODYN | |
| --- | --- | --- | --- | --- | --- |
| # | Variable | Mean | SD | Mean | SD |
| 1 | AMF biomass | 5.21 | 2.14 | 12.04 | 6.71 |
| 2 | Bacterial biomass | 30.84 | 5.57 | 42.95 | 3.03 |
| 3 | Fungal biomass | 1.05 | 0.21 | 1.40 | 0.20 |
| 4 | Microbial nitrogen (N) | 41.56 | 9.08 | 80.30 | 4.68 |
| 5 | Microbial carbon (C) | 281.88 | 53.99 | 484.61 | 29.69 |
| 6 | Nematoda abundance | 1229.39 | 997.09 | 1153.31 | 610.60 |
| 7 | Collembola abundance. | 7324.44 | 14908.57 | 12060.44 | 16968.96 |
| 8 | Oribatida abundance. | 1813.00 | 1576.27 | 2864.56 | 3371.27 |
| 9 | Chilopoda abundance. | 53.67 | 49.81 | 56.78 | 59.30 |
| 10 | Diplopoda abundance. | 71.56 | 129.35 | 99.56 | 238.66 |
| 11 | Araneae activity density | 10.28 | 5.06 | 12.25 | 6.44 |
| 12 | Staphylinidae activity density | 7.61 | 7.12 | 4.75 | 7.25 |
| 13 | Arable weed cover | 1.39 | 4.42 | 29.89 | 25.91 |
| 14 | Bacterial diversity | 6.77 | 0.06 | 6.79 | 0.07 |
| 15 | Nematoda diversity | 1.95 | 0.26 | 1.94 | 0.22 |
| 16 | Soil mesofauna diversity | 0.64 | 0.29 | 0.69 | 0.31 |
| 17 | Soil macrofauna diversity | 1.10 | 0.35 | 1.03 | 0.44 |
| 18 | Araneae diversity | 1.23 | 0.64 | 1.49 | 0.53 |
| 19 | Microbial respiration | 0.40 | 0.14 | 0.55 | 0.17 |
| 20 | Soil feeding activity | 44.57 | 31.96 | 54.55 | 28.91 |
| 21 | Litter decomposition | 80.73 | 17.61 | 79.73 | 14.13 |
| 22 | Soil water content | 16.25 | 5.64 | 18.67 | 6.28 |
| 23 | Soil mineral N | 9.71 | 9.36 | 4.80 | 1.19 |
| 24 | C content wheat | 44.19 | 0.86 | 43.38 | 0.75 |
| 25 | N content wheat | 2.21 | 0.66 | 1.86 | 0.79 |
| 26 | Total wheat biomass | 9.79 | 6.00 | 10.48 | 5.61 |

**Supplementary Table s2** Means and standard deviation of individual factor levels of fixed factor “Time” and all 26 dependent variables.

|  |  | T1 | | T2 | | T3 | |
| --- | --- | --- | --- | --- | --- | --- | --- |
| # | Variable | Mean | SD | Mean | SD | Mean | SD |
| 1 | AMF biomass | 5.83 | 1.60 | 7.08 | 2.98 | 12.97 | 8.38 |
| 2 | Bacterial biomass | 38.58 | 7.25 | 36.73 | 8.09 | 35.36 | 7.25 |
| 3 | Fungal biomass | 1.33 | 0.27 | 1.19 | 0.24 | 1.17 | 0.27 |
| 4 | Microbial nitrogen (N) | 62.47 | 23.43 | 61.78 | 20.03 | 60.28 | 19.68 |
| 5 | Microbial carbon (C) | 404.09 | 113.19 | 376.83 | 110.00 | 379.00 | 111.89 |
| 6 | Nematoda abundance | 1063.30 | 501.18 | 921.86 | 511.58 | 1588.88 | 1146.34 |
| 7 | Collembola abundance. | 14613.33 | 19022.94 | 14293.33 | 17029.99 | 170.67 | 326.16 |
| 8 | Oribatida abundance. | 2007.83 | 1742.42 | 1731.33 | 1092.16 | 3277.17 | 4036.66 |
| 9 | Chilopoda abundance. | 74.67 | 53.93 | 72.33 | 55.34 | 18.67 | 32.68 |
| 10 | Diplopoda abundance. | 149.33 | 294.32 | 85.17 | 127.77 | 22.17 | 36.91 |
| 11 | Araneae activity density | 13.92 | 5.69 | 6.29 | 2.82 | 13.58 | 5.11 |
| 12 | Staphylinidae activity density | 3.00 | 2.77 | 5.21 | 4.08 | 10.33 | 10.47 |
| 13 | Arable weed cover | 25.58 | 29.35 | 19.58 | 22.36 | 1.75 | 2.21 |
| 14 | Bacterial diversity | 6.75 | 0.04 | 6.74 | 0.05 | 6.85 | 0.05 |
| 15 | Nematoda diversity | 1.95 | 0.26 | 1.99 | 0.21 | 1.88 | 0.24 |
| 16 | Soil mesofauna diversity | 0.82 | 0.19 | 0.72 | 0.23 | 0.45 | 0.34 |
| 17 | Soil macrofauna diversity | 1.22 | 0.34 | 1.09 | 0.35 | 0.90 | 0.43 |
| 18 | Araneae diversity | 1.33 | 0.63 | 1.05 | 0.64 | 1.71 | 0.29 |
| 19 | Microbial respiration | 0.46 | 0.11 | 0.60 | 0.18 | 0.35 | 0.10 |
| 20 | Soil feeding activity | 26.81 | 19.32 | 69.84 | 27.40 | 52.02 | 28.37 |
| 21 | Litter decomposition | 79.41 | 15.57 | 84.97 | 15.25 | 76.71 | 16.26 |
| 22 | Soil water content | 17.49 | 2.86 | 23.40 | 5.04 | 11.48 | 2.32 |
| 23 | Soil mineral N | 12.57 | 9.83 | 4.76 | 1.68 | 4.43 | 3.27 |
| 24 | C content wheat | 43.24 | 0.74 | 44.11 | 0.68 | 44.00 | 1.01 |
| 25 | N content wheat | 2.86 | 0.21 | 2.05 | 0.38 | 1.19 | 0.27 |
| 26 | Total wheat biomass | 4.76 | 1.25 | 7.91 | 1.18 | 17.74 | 2.08 |

**Supplementary Table s3** Means and standard deviation of individual factor levels of fixed factor “Drought” and all 26 dependent variables.

|  |  | Roof | | Roof Control | | Control | |
| --- | --- | --- | --- | --- | --- | --- | --- |
| # | Variable | Mean | SD | Mean | SD | Mean | SD |
| 1 | AMF biomass | 10.58 | 7.34 | 8.19 | 5.91 | 7.10 | 4.06 |
| 2 | Bacterial biomass | 36.96 | 8.57 | 36.74 | 7.42 | 36.98 | 6.90 |
| 3 | Fungal biomass | 1.23 | 0.28 | 1.23 | 0.32 | 1.22 | 0.21 |
| 4 | Microbial nitrogen (N) | 60.64 | 19.46 | 61.37 | 22.36 | 62.47 | 21.30 |
| 5 | Microbial carbon (C) | 387.25 | 109.15 | 381.61 | 116.22 | 389.52 | 111.55 |
| 6 | Nematoda abundance | 1426.26 | 1077.31 | 967.56 | 474.99 | 1180.22 | 767.21 |
| 7 | Collembola abundance. | 8448.00 | 15506.96 | 9002.67 | 16083.45 | 11626.67 | 16994.19 |
| 8 | Oribatida abundance. | 1752.33 | 1537.57 | 2177.00 | 1701.04 | 3087.00 | 3964.04 |
| 9 | Chilopoda abundance. | 57.17 | 65.26 | 47.83 | 44.83 | 60.67 | 52.65 |
| 10 | Diplopoda abundance. | 108.50 | 290.43 | 86.33 | 147.91 | 61.83 | 73.38 |
| 11 | Araneae activity density | 10.63 | 4.24 | 11.58 | 6.29 | 11.58 | 6.85 |
| 12 | Staphylinidae activity density | 6.75 | 7.85 | 7.83 | 9.05 | 3.96 | 3.38 |
| 13 | Arable weed cover | 14.67 | 24.47 | 15.00 | 22.87 | 17.25 | 23.68 |
| 14 | Bacterial diversity | 6.80 | 0.06 | 6.77 | 0.08 | 6.77 | 0.06 |
| 15 | Nematoda diversity | 1.90 | 0.22 | 1.96 | 0.23 | 1.96 | 0.27 |
| 16 | Soil mesofauna diversity | 0.64 | 0.30 | 0.65 | 0.33 | 0.70 | 0.29 |
| 17 | Soil macrofauna diversity | 1.06 | 0.41 | 1.05 | 0.40 | 1.10 | 0.38 |
| 18 | Araneae diversity | 1.45 | 0.56 | 1.32 | 0.61 | 1.32 | 0.64 |
| 19 | Microbial respiration | 0.41 | 0.19 | 0.49 | 0.16 | 0.53 | 0.13 |
| 20 | Soil feeding activity | 25.76 | 17.21 | 57.33 | 26.68 | 65.59 | 31.21 |
| 21 | Litter decomposition | 79.42 | 13.96 | 77.69 | 17.89 | 83.51 | 15.41 |
| 22 | Soil water content | 13.84 | 3.77 | 18.12 | 6.05 | 20.42 | 6.24 |
| 23 | Soil mineral N | 6.58 | 4.34 | 7.63 | 7.95 | 7.55 | 8.47 |
| 24 | C content wheat | 43.86 | 0.95 | 43.75 | 0.88 | 43.74 | 0.91 |
| 25 | N content wheat | 2.02 | 0.74 | 2.04 | 0.77 | 2.04 | 0.76 |
| 26 | Total wheat biomass | 10.12 | 5.88 | 10.49 | 5.90 | 9.79 | 5.77 |

**Supplementary information**

*Sampling methods and approaches*

**Bacterial, fungal and AMF biomass** (variables 1-3 in Tab. 2) were derived from phospholipid fatty acid analysis according to Frostegård et al. (1993) as described in Kundel et al. (2020). In brief, fatty acid methyl esters were separated by gas chromatography (Hewlett Packard 6890, Palo Alto, USA). Bacterial abundance was then derived from summing abundances of phospholipid fatty acids i15:0, a15:0, 15:0, i16:0, 16:1ω9, i17:0, a17:0, cy17:0, 18:1ω7, 20:0 and cy19:0 (Frostegård and Bååth, 1996), the amount of PLFA 18:2ω6 served as indicator markers for non-mycorrhizal fungi while the neutral lipid fatty acid (NLFA) 16:1ω5 for arbuscular mycorrhizal fungi.

**Microbial biomass nitrogen (Nmic) and carbon (Cmic)** (4-5) were determined by chloroform-fumigation extraction (CFE) following Vance et al. (1987). Directly before extraction, we adjusted soil moisture to 85% dry matter content and extracted soil samples in 0.5 M K_2_SO_4_ with and without prior chloroform fumigation. Soil suspensions were filtered (Filter: MN 615 ¼, Macherey-Nagel, Düren, Germany) and Cmic and Nmic values measured and calculated as described in Fliessbach et al. (2007).

**Nematoda abundance** (6) was counted based on Nematodes extracted from 200 g of fresh soil by a modification of the Baermann funnel method after a decanting and wet sieving process (Barker, 1985). All nematodes were counted under a stereomicroscope and nematode numbers were reported as number of individuals per 100 g of dry soil.

**Collembola abundance** (7) was counted based on Collembola extracted from soil cores with a diameter of 5 cm and a depth of 10 cm with heat gradient extraction by increasing temperature from 25 to 55°C in steps of 2.5°C until 30°C and in steps of 5°C from 30 to 55°C (Macfadyen 1961).

**Oribatida, Chilopoda & Diplopoda abundance** (8-10) was counted based on individuals extracted from soil cores with a diameter of 20 cm and a depth of 10 cm with heat gradient extraction by increasing temperature from 25 to 55°C in steps of 5°C (Kempson et al. 1963); Oribatida abundances were extrapolated from the larger cores instead of the small cores (as typical for mesofauna), because densities were very low.

**Araneae & Staphylinidae activity density** (11&12) were counted from one pitfall trap (55mm ø) in each subplot activated for 14 days before each sampling time (T1-T3). Activity-density denotes the total number of individuals caught by each trap in the 14-day period (Greenslade, 1964).

**Arable weed cover** (13) was derived from visually estimating the proportion of soil surface covered by arable weeds (Lotz et al., 1994).

**Bacterial diversity** (14) was derived from Amplicon sequencing of 16S rRNA marker genes. For PCR cycling conditions, selected oligonucleotides and sequencing (Kundel et al., 2020). The composition of the whole community was used to calculate the Shannon diversity index (to the base e). The sequencing data underlying this article are available in the NCBI Sequence Read Archive (SRA) database at https://www.ncbi.nlm.nih.gov/bioproject and can be accessed with the study accession number BioProject ID PRJNA641521

**Nematoda diversity** (15) was derived by mounting 150 nematodes on temporary in each sample, and then identifying these individuals to genera or family level. The taxonomic composition of the whole nematode community was used to calculate the Shannon diversity index (to the base *e*).

**Soil meso- and macrofauna diversity** (16&17) were calculated as the Shannon diversity index (to the base *e*) based on the counts of the three groups Collembola, Oribatida and Acari (excl. Oribatida) for meso- and the seven groups Araneae, Carabidae, Chilopoda, Diplopoda, Hymenoptera, Isopoda and Staphylinidae for macrofauna.

**Araneae diversity** (18) was calculated as the Shannon diversity index (to the base *e*) based on species-level identification of all adult specimens caught in the pitfall traps for activity-density estimates.

**Microbial respiration** (19) was measured according to Jäggi (1976) as CO_2_ evolution after pre-incubating soils for seven days at 22°C without adjustment of soil water contents. Soils were sieved (2 mm) and stored at 4°C before analysis.

**Soil feeding activity** (20) was assessed by means of bait-lamina strips, each consisting of a PVC strip (120 mm × 5 mm × 1 mm) including along its length sixteen 1.5 mm ø holes separated 5 mm from each other (Kratz 1998). Each hole was filled with bait powder composed of a mixture of cellulose, bran flakes and active coal (70:27:3), which was moistened with tap water till obtaining a cement-like texture. Filling of holes was repeated 3 times to minimize the presence of bait gaps inside the holes. Fourteen days before each sampling date (T1-T3), nine bait-laminas were equidistantly inserted in the soil of each experimental plot in a 3 × 3 (20×20 cm) matrix. Strips were inserted till the top hole was just below the soil surface. At each sampling date, bait-laminas were removed, carefully wrapped with plastic-film, and stored at 4°C. Feeding activity was assessed in the lab under a dissection microscope, using a five-point scale, with points corresponding to the approximate percentage of the area of bait consumed: 0 – no consumption; 25 – consumed about 25%; 50 – about 50%; 75 – about 75%; and 100 – fully empty.

**Litter decomposition** (21) was estimated with three litterbags per subplot that were installed between wheat stands and fixed to the soil surface with tent poles 14 days before the first sampling date (T1). At each sampling date one randomly selected bag was collected and further processed. Litter-bags (1 mm mesh size) contained 5 g of oven-dried litter (60°C for 72 h). The remaining litter was oven-dried at 60°C for 72 h and weighed to the nearest 0.01 g. Lastly, initial and final C and N contents were estimated in 3 mg subsamples using an elementar analyzer (Vario EL III, Elementar Analysesysteme, Hanau, Germany). Finally, mineralization rate was calculated as the C:N ratio.

**Soil water content** (22) was assessed from 100 g of field moist soil that was oven-dried at 105°C to constant weight. Soil water content is expressed in terms of the mass of water per unit mass of the dry soil.

**Soil mineral N** (23) was extracted from sieved (2 mm), field moist soil samples in 0.01 M CaCl_2_ solution (1:4 w/v) by shaking samples vigorously for 1 hour. The soil suspension was filtered (MN 619EH; Macherey-Nagel, Düren, Germany) followed by photospectrometrical determination of ammonium and nitrate (SAN-plus Segmented Flow Analyzer; Skalar Analytical B.V., Breda, Netherlands).

**C and N content shoot** (24&25) Carbon (C) and nitrogen (N) concentrations in the plant (total aboveground material) was assessed from 10 mg dried and ball-milled material (10 mg) on a CN analyzer (Vario EL III, Elementar Analysensysteme GmbH, Langenselbold, Germany)

**Crop biomass production** (26) was assessed from wheat biomass that was harvested from 20 cm × 50 cm areas in each subplot (two wheat rows), cut to smaller pieces and dried at 60°C to constant weight.

**References**

Barker, K. R. (1985). Nematode extraction and bioassays. An advanced treatise on Meloidogyne, 2, 19-35.

Fließbach, A., Oberholzer, H. R., Gunst, L., & Mäder, P. (2007). Soil organic matter and biological soil quality indicators after 21 years of organic and conventional farming. Agriculture, Ecosystems & Environment, 118(1-4), 273-284.

Frostegård, Å., Tunlid, A., & Bååth, E. (1993). Phospholipid fatty acid composition, biomass, and activity of microbial communities from two soil types experimentally exposed to different heavy metals. Applied and Environmental Microbiology, 59(11), 3605-3617.

Greenslade, P. J. M. (1964). Pitfall trapping as a method for studying populations of Carabidae (Coleoptera). The Journal of Animal Ecology, 301-310.

Jäggi, W. (1976). Die Bestimmung der CO2-Bildung als Maß der bodenbiologischen Aktivität. Schweizer Landwirtschaftliche Forschung, 15, 371-380.

Kempson, D., Lloyd, M., & Ghelardi, R. (1963). A new extractor for woodland litter. Pedobiologia, 3(1), 1-21.

Kratz, W. (1998). The bait-lamina test. Environmental Science and Pollution Research, 5(2), 94-96.

Kundel, D., Bodenhausen, N., Jørgensen, H. B., Truu, J., Birkhofer, K., Hedlund, K., ... & Fliessbach, A. (2020). Effects of simulated drought on biological soil quality, microbial diversity and yields under long-term conventional and organic agriculture. FEMS microbiology ecology, 96(12), fiaa205.

Lotz, L. A. P., Kropff, M. J., Wallinga, J., Bos, H. J., & Groeneveld, R. M. W. (1994). Techniques to estimate relative leaf area and cover of weeds in crops for yield loss prediction. Weed Research, 34(3), 167-175.

Macfadyen, A. (1961). Improved funnel-type extractors for soil arthropods. The Journal of Animal Ecology, 171-184.

Vance, E. D., Brookes, P. C., & Jenkinson, D. S. (1987). Microbial biomass measurements in forest soils: determination of kc values and tests of hypotheses to explain the failure of the chloroform fumigation-incubation method in acid soils. Soil Biology and Biochemistry, 19(6), 689-696.
